# Successful Treatment of Essential Palatal Tremor Lasting Over a Long Term with a Rare Application of Botulinum Toxin in a Child

**Published:** 2015

**Authors:** Aylin Eryılmaz, Yeşim Başal, Ceren Günel, Ayca Ozkul, Ayşe Tosun

**Affiliations:** 1 Department of Otorhinolaryngology Head and Neck Surgery, Adnan Menderes University School of Medicine, Aydın, Turkey; 2 Department of Neurology Adnan Menderes University School of Medicine, Aydın, Turkey; 3 Department of Pediatric Neurology, Adnan Menderes University School of Medicine, Aydın, Turkey

**Keywords:** Botulinum toxin, Child, Essential palatal tremor

## Abstract

**Objective**

Essential Palatal Tremor (PT) is a disorder in which radiological brain images appear normal but the clicking noise caused by peritubal, palatal muscle contractions remains the main complaint of patients. The condition occurs rarely in childhood. This paper demonstrates such a rare case with bilateral presentation of essential PT in a 12-yr-old girl could successfully be treated with botulinum toxin therapy at Otorhinolaryngology Department in 2013, as she was still asymptomatic after 16 months. Besides, being minimally invasive with negligible side effects, this choice of treatment with botulinum toxin A (BTA) leads to a long-term symptom free outcome from essential PT.

## Introduction

Palatal tremor (PT) is a neurotologic disorder with essential and secondary forms (1). Essential PT is a very annoying benign situation usually disappearing spontaneously or during asleep, whereas secondary PT is frequently unilateral and persists even during sleep (1). In essential PT, brain images appear normal, but patients complain of a clicking noise caused by the rapid opening and closing of the eustachian tube due to the contractions of musculus levator and tensor veli palatinis (1). Several options like anticonvulsants, anxiolytics and botulinum toxin A (BTA) with varying degrees of therapeutic efficacies are available for treating this condition (2, 3). BTA treatment in general offers safety, minimal invasiveness, patient comfort and negligible side effects opposed to the other alternatives (3). However, so far, it was rarely applied in children. Here we demonstrate that its use is better suited for the successful treatment of essential PT seen rarely in children.

## Case Report

A 12 yr old girl with a clicking tinnitus heard externally was presented at Otorhinolaryngology Department in 2013. She was complaining of not hearing her teacher in the classroom because of this condition that persisted for about a year. In general, otorhinolaryngological examination, the clicks could be heard easily by the examiner at a 20 cm distance from the patient and the frequency of the clicks was 60-80 per min. Further physical examination showed an objective constriction of the palatal muscles bilaterally. There was no other accompanying symptom. Thus, this led to the diagnosis of PT. Her systemic and neurological examinations were normal. The audiologic test was within the normal range. Magnetic resonance imaging (MRI) indicated no pathological findings. Routine laboratory results indicated no abnormality. The psychiatric evaluation was clear. These findings decisively supported the existence of essential PT. The parents of the patient were discussed about the available treatment options for the essential PT such as wait and see, anticonvulsants, sedatives and BTA injection. Antiepileptic drugs were also included in the discussion by mentioning their common side effects like dizziness, drowsiness, mental slowing down, weight gain, nephrolithiasis, skin rash, hepatotoxicity and behavioral disorders. At the end, besides the primary request of the parents not to disturb the patient’s educational activities and considering its safety, minimal invasiveness, patient comfort and negligible side effects opposed to the other alternatives, BTA injection was decided to be the best treatment option for this case. The parents were taken written informed consent on this procedure. Five hundred mouse units (MU) of BTA (Dysport®) were dissolved in 5 mL NaCl 0.9% solution. Only a topical anesthesia with tetracain spray was used. 0.3 Ml of this solution (corresponding to the amount of 30 MU) was injected with a dental syringe in the right muscle tensor and levator veli palatinis and 30 MU to the left muscle tensor and levator veli palatinis. Regular telephone interviews were conducted. There was no palatal myoclonus on the 5 days follow up period. However, the patient complained of a regurgitation of fluids and deformation of her speech for 3 days. By the time of first control examination, these complaints were disappeared. In another examination after16 months, she was still free of palatal tremor even after such a long time since such tremors reoccur within few months of the initial treatment and may require repetitive treatments.

## Discussion

There are limited numbers of publications reporting the occurrence of essential PT in pediatric population (3-10). Because of the low numbers in child essential PT, there is little experience in therapeutic efforts. Botulinum toxin treatment in essential PT, to date, has been reported in 8 children (3,7-12). Jamieson et al. reported a 17 yr old boy with left essential PT (12). They used 44 MU (botox ®) botulinum toxin and the symptom resolved. Jero et al. also treated a 12 yr old girl with right palatal tremor. Her symptoms resolved with 20 MU (Dysport®) botulinum toxin injection (7). Ensink et al. reported an 8 yr old boy with left palatoclonus treated by botulinum toxin 3 times in a 18 month period. The total botulinum dose was 60 MU ( Dysport®). The side effects were pain in injection and slight limitation in swallowing. Beside psychological problems the clicking became severe. The eustachian tube was occluded two times. The clicks after that disappeared (9). In our case, there was no pain in injection but the patient complained of a regurgitation of fluids and deformation of her speech for 3 days. By the time of first control examination, these complaints were disappeared. A 10 yr old girl with left essential PT was treated with 30 MU BTA (Dysport®) into the tensor veli palatini muscle. No immediate side effects were noted. On the 7th day the clicking tinnitus was neither noticeable nor palatal contractions were longer present. The patient was symptom free for 4 years (8, 11). In our case the same dose of BTA was applied. She had no palatal contactions on the 5th day and was symptom free for 16 months. Pal PK et al. reported an 8 yr old boy with bilateral essential PT (10). His complains had no response to medications. 4 IU BTA (Botox®, Allergan) was injected to each tensor veli palatine muscle. He had only ear pain in the first 24 h. He had only fluid regurgitation on the second day but our case had 3 days regurgitation. He was symptom free on the 6th day. After 3 months, his palatal tremors began and on the first year the BTA injection with 5 IU BTA (Botox®, Allergan) to each tensor veli palatine muscle is repeated. He had complete palatal palcy on the 5th day and pain in swallowing which required feeding from nasogastric tube and after one month, his swallowing was normal. He had a 5 month symptom free follow up (10). Carman et al. reported a 9 yr old boy with a 2 yr history of PT (3). The treatments like lamotrigine, levetiracetam and clonazepam failed in this case. 2.5 IU BTA (Botox®, Allergan) was injected in two different parts of tensor veli palatine muscle. He had mild regurgitation which resolved in one week. He was symptom free within 2 weeks and is symptom free for 10 months since the article is written (3). Krause et al. made botulinum toxin injection in essential PT child in a 6 yr old boy. The same dose of Dysport®was made to the right soft palate. On the second day, the symptoms disappeared but after 24 weeks, the symptoms reoccurred bilaterally. Fifty MU Dysport® was made bilaterally. This patient is symptom free for two years (11). In our case, there was bilateral essential PT therefore; we injected 30 MU BTA (Dysport®) to the right and 30 MU BTA to the left muscle tensor and levator veli palatinis. The dose of BTA in children is often 30 MU but we see that also 50 MU can be tolerated by children without side effects. With a dose-dependent reduction of abnormally increased muscle or secretory activity, diseases of various etiologies can be treated with botulinum toxin. The disadvantage is the need for repeat in application but we see that in child group essential PT there is mostly one application enough. The BTA treatment lasted with a long time treatment successes in children (3, 8, 11). This favorable outcome could not be explained with the local effect of BTA. Instead, a possible explanation would be the disruption of peripherally triggered central reentry mechanism, leading to the temporary paralysis of peripheral muscle. There are also medical treatments like piracetam in child PT (13) which are also successful but these medications can have side effects. In this report, four children with essential PT had piracetam and the symptoms disappeared within 20 days. In one patient the medicationwas stopped, and this coincided with the reappearanceof tremor. Reintroduction of piracetam was rapidly effective in recontrolling the tremor (13). The long lasting therapeutic effect from botulinum toxin in essential PT remains speculative. The etiology and pathophysiology of essential PT needs more studies. The clinical results of botulinum toxin treatment in essential PT in children are very successful and safe but further studies with larger numbers of patients are needed.

## Notes

**Authors’ contribution** AE: design, analysis, writing, final approval;

YB: analysis, writing; CG: revising, analysis;

AO: revising, design; AT: analysis, writing, final approval
